# MiR-4319 induced an inhibition of epithelial-mesenchymal transition and prevented cancer stemness of HCC through targeting FOXQ1

**DOI:** 10.7150/ijbs.38000

**Published:** 2019-11-15

**Authors:** Shaoshan Han, Yu Shi, Liankang Sun, Zhikui Liu, Tao Song, Qingguang Liu

**Affiliations:** 1 Department of Hepatobiliary Surgery, the First Affiliated Hospital of Xi'an Jiaotong University, No. 277 Yanta West Road, Xi'an 710061, China; 2 Department of oncology, the First Affiliated Hospital of Xi'an Jiaotong University, No. 277 Yanta West Road, Xi'an 710061, China

**Keywords:** miR-4319, FOXQ1, EMT, cancer stemness, HC

## Abstract

The heterogeneity existing in tumours is responsible for the poor response to treatment. Therefore, elucidating the molecular mechanisms of intratumoural heterogeneity in hepatocellular carcinoma (HCC) is vital for the discovery of new therapeutic methods for improving the prognosis of patients. Of note, cancer stem cells (CSCs) existing in HCC may explain the pathological properties of heterogeneity and recurrence. An increasing number of studies have confirmed that abnormally expressed microRNAs (miRNAs) take part in the carcinogenesis as well as the aggravation of HCC. However, little information is currently available about the specific miR-4319 in HCC. Herein, we demonstrated that the level of miR-4319 was remarkably decreased in HCC specimens and cells compared to that in normal counterparts and that the depression of miR-4319 in tumour specimens correlates with tumour size, histological grade and venous invasion. Through a series of functional experiments, we illustrated that miR-4319 repressed cell proliferation, accelerated apoptosis, inhibited epithelial-mesenchymal transition (EMT) and prevented cancer stemness in HCC cells by targeting FOXQ1 (Forkhead box Q1). An *in vivo* tumourigenesis assay uncovered that depletion of miR-4319 in Hep3B cells increased tumour growth and elevated the expression of EMT and CSC markers in comparison to those of the control group. Restoration of FOXQ1 expression also partially reversed the miR-4319-induced biological effects on HCC cells. Thus, miR-4319, as a posttranscriptional regulator, plays a profound role in suppressing the malignant progression of HCC, and our study highlights the miR-4319/FOXQ1 cascade as a potential therapeutic target for conquering HCC.

## Introduction

Hepatocellular carcinoma (HCC) is one of the most aggressive malignancies in the world 1, displaying a dismal prognosis due to the high heterogeneity and high rate of recurrence and metastasis 2, 3, 4. Although surgical resection is the optimal choice for early-stage HCC, most patients suffering from HCC are often diagnosed at an advanced stage 5. Therefore, it is imperative to investigate the underlying molecular mechanism participating in the initiation and malignant progression of HCC and urgent to discover new therapeutic targets for HCC patients.

Notably, recent investigations have uncovered that HCC has a heterogeneous cell population named cancer stem cells which display stemness features that are pivotal for tumourigenesis, distant dissemination and chemoresistance of HCC 6-8. Hepatocellular carcinoma harbours a population of cancer stem cells or possesses the high expression of a panel of genes with stemness markers, which may be a signal for predicting poor treatment outcomes for HCC patients 9. Moreover, cancer stem cells have gradually become promising therapeutic targets of HCC 6, 10-12, which indicates that finding novel genes modulating cancer stemness could elevate the treatment outcome of HCC. Epithelial-mesenchymal transition (EMT) has been identified as a vital process in the early metastatic dissemination of cancer cells by endowing them with a more invasive phenotype 13. Intriguingly, a tight link exists between acquiring cancer stem-like traits and EMT induction 14. EMT induction in mammary epithelial cells generates populations of cells that possess cancer stem-like traits, as determined by mammosphere formation, tumour-seeding capacity, and expression of surface marker profiles of stemness 14, 15. In addition, CSCs are prone to elevate the expression of multiple factors involved with mesenchymal transdifferentiation, such as vimentin fibronectin and EMT-related transcription factors 14.

As a set of evolutionarily conserved small non-coding RNAs, microRNAs (miRNAs) have been identified as post-transcriptional regulators at the level of gene expression, which is achieved by the combination of complementary sequences with the 3'-untranslated regions (UTRs) of corresponding mRNA of genes encoding for proteins, leading to mRNA degradation or translation cessation^16^, ^17^. A growing number of studies have indicated that miRNAs are vital in diverse biological processes in HCC, including cell proliferation, the cell cycle, apoptosis, differentiation, metastasis and cancer stemness, by regulating tumour suppressors or oncogenes 18, 19. Therefore, specific miRNAs are known to be potential therapeutic, diagnostic, and prognostic biological markers in HCC 20. MiR-4319, a newly identified cancer-related microRNA, was aberrantly expressed and became a predictor of patient survival in cancers 21. Previous studies have revealed that miR-4319 elicits inhibitory effects on the proliferation of breast cancer and prostate cancer 22, 23, and miR-4319 could repress cancer stemness in triple-negative breast cancer through targeting E2F2 22. MiR-4319 also exhibits tumour suppressor activities in NSCLC (non-small-cell lung cancer) through targeting LIN28/RFX5/YAP cascades to mitigate cell migration and proliferation and facilitate cell apoptosis 24. Moreover, miR-4319 is transcriptionally modulated by PLZF and acts as a tumour suppressor in CRC (colorectal cancer) via targeting ABTB1 21. MiR-4319 suppresses the development of thyroid cancer by regulating FUS (fused in sarcoma)-stabilized SMURF1 (SMAD-specific E3 ubiquitin protein ligase 1) 25. Nevertheless, the biological effects and the latent regulatory mechanism of miR-4319 in HCC remain obscure and need to be further elucidated. The current study investigated the effects and modulatory mechanism of miR-4319 in HCC.

Herein, we uncovered that miR-4319 was reduced in HCC and correlated with adverse prognostic features. Moreover, miR-4319 impeded cell proliferation, accelerated apoptosis, inhibited epithelial-mesenchymal transition and prevented cancer stemness of HCC through targeting FOXQ1.

## Materials and Methods

### HCC specimens and cell culture

In total, 83 HCC specimens and the corresponding neighbouring non-tumour samples were gathered in our hospital from January 2008 to December 2011 with informed consent from all patients. None of the patients had received any prior radiotherapy or chemotherapy. This study was approved by the Ethics Committee of the First Affiliated Hospital of Xi'an Jiaotong University, according to the Declaration of Helsinki.

The human immortalized normal hepatocyte cell line LO2 and a group of HCC cell lines (HepG2, Hep3B, SMMC-7721, MHCC-97L and MHCC-97H) (Chinese Academy of Sciences, Shanghai, China) were cultured in DMEM (Invitrogen, Carlsbad, USA) with 10 μl of 10% FBS (Gibco, Grand Island, USA) and incubated at 37 °C and 5% CO_ 2_.

### RNA extraction and qRT-PCR

Total RNA in the tissue samples and HCC cell lines was extracted using TRIzol reagent. The quantity and quality of isolated total RNA were evaluated as previously reported 26. The primers used for qRT-PCR are displayed as follows: miR-4319 (forward) 5′-GCACAGCTCCCTGAGCAA-3′ and (reverse) 5′-CAGTGCGTGTCGTGGAGT-3′; U6 (forward) 5′-CTCGCTTCGGCAGCACAT-3′ and (reverse) 5′-TTTGCGTGTCATCCTTGCG-3′; and FOXQ1 (forward) 5′-GATTTCTTGCTATTGACCGATGC-3′ and (reverse) 5′-CTAATAAAGCTGTAGCCCGTTGC-3′. The primers against GAPDH (HQP006940) were obtained from Genecopoeia (Guangzhou, China). qRT-PCR was conducted as reported previously^27^. The 2^-ΔΔCt^ method was utilized to calculate the relative gene expression.

### Cell transfection

MiR-4319 mimic (#4464066), miR-4319 inhibitor (#4464084) or corresponding negative control oligonucleotides (NC mimic and NC inhibitor) were all obtained from Invitrogen. The validated small interfering RNA (siRNA) targeting FOXQ1 and negative control siRNA were purchased from Ambion as previously reported 28. The FOXQ1 expression plasmid (pcDNA3.1-FOXQ1) was purchased from GenePharma (Shanghai, China). HCC cells were transfected with oligonucleotides and vectors using Lipofectamine 2000 (Invitrogen) based on the manufacturer's instructions. Cells were harvested 48 h post-transfection for further experiments.

### Western blot analysis

RIPA buffer (Beyotime, Shanghai, China) was used to lyse the total protein derived from cultured HCC cells. Protein concentrations were determined using a BCA protein assay kit (Pierce Biotechnology, Inc., Rockford, IL, USA). Equal amounts of protein (20 μg/lane) were separated on SDS-PAGE and transferred onto a PVDF membrane (Bio-Rad Laboratories, Hercules, CA, USA). The primary antibodies used in this study are displayed in Supplementary Table 1. The membranes were incubated with horseradish peroxidase (HRP)-conjugated secondary antibodies (NXA931-1ML and NA934-1ML, GE Healthcare Life Sciences, Beijing, China). Protein bands were visualized using an enhanced chemiluminescence detection kit (Pierce Biotechnology). Blots were semi-quantified by ImageJ software (1.46; National Institutes of Health, Bethesda, MD, USA).

### Colony formation assay

After completing the designated intervention, Hep3B and MHCC-97H cells (1000 cells per well) were seeded in a 6-well plate and cultured for two weeks. Then, the colonies were fixed with 4% paraformaldehyde and stained with crystal violet solution after washing three times with PBS. The number of visible colonies was counted under a microscope.

### MTT assay

The cell viability of HCC cells was determined by 3-(4,5-dimethylthiazol-2-yl)-2,5-diphenyl tetrazoliumbromide (MTT) assays. HCC cells were seeded in a 96-well plate at a density of 5×10^3^ cells/well and transfected with miR-4319 mimics, miR-4319 inhibitor, pcDNA3.1-FOXQ1 and si-FOXQ1 for 24, 48, 72, or 96 h. Then, 10 μl of MTT (Sigma‑Aldrich; Merck KGaA) was administered to cultured cells and incubated for 4 h at 37˚C. The supernatant was replaced with 100 μl of DMSO incubated for 15 min, and the absorbance was determined at 490 nm using a multiwell microplate reader.

### EdU assay

An ethynyl deoxyuridine (EdU) incorporation assay was conducted with an EdU kit according to the manufacturer's protocols. The images of EdU staining were visualized by a Zeiss fluorescence photomicroscope (Carl Zeiss, Oberkochen, Germany) and quantified via counting at least five random fields.

### Transwell invasion assay

After completing the designated transfection, HCC cells were resuspended with serum-free medium and seeded into the upper chamber coated with Matrigel (Sigma). Culture medium containing 10% FBS served as a chemoattractant in the lower chamber. After being cultured for 48 hours at 37°C, cells that did not invade via the pore of the filter were carefully wiped out with cotton wool. Then, the invasive cells were fixed with 100% methanol and stained with crystal violet.(Sigma). Cell number was counted under a microscope by randomly selecting 5 fields.

### Tumoursphere formation assay

HCC cells transfected with miR-4319 mimics, miR-4319 inhibitor, pcDNA3.1-FOXQ1 and si-FOXQ1, were plated in 6‑well ultra‑low-attachment plates (Corning Incorporated, Corning, NY, USA) at a density of 5×10^3^ cells/well in serum‑free DMEM/F12 medium. The DMEM/F12 medium was supplemented with 1% B27, 20 ng/ml human EGF and 20 ng/ml human FGF. Cells were subsequently cultured at 37˚C for 2 weeks to allow formation of tumourspheres. After 14 days, tumourspheres were counted under a light microscope (Nikon Corporation) at a magnification of ×200, and the number was recorded.

### Dual-luciferase reporter assay

The 3'-UTR binding sequence of FOXQ1 and the associated mutated sequences were generated and inserted into the pmiR-GLO dual luciferase miRNA target expression vector (Promega, Madison, WI, USA). The dual-luciferase reporter assay was performed according to previous instructions 18.

### *In vivo* tumourigenesis assay

A 4-to-6-week-old female BALB/c nude mouse (obtained from the Experimental Animal Center of Xi'an Jiaotong University School of Medicine, Xi'an) was utilized to establish a subcutaneously implanted tumour model. The xenograft tumours were generated using the Hep3B cell line stably depleting miR-4319 or its corresponding controls. The stable miR-4319-depleting Hep3B cells were generated by infection with lentiviral vector based on the manufacturer's instructions (miR-4319: pLV-[hsa-mir-4319] plasmid; negative control plasmid: pLV-[mir-control], Biosettia), which were in accord with previously described methods 28. After establishing a stable expression cell line, 5×10^6^ cells were mixed into 150 μL of Matrigel and injected subcutaneously into the flanks of nude mice. The tumour volume was then monitored by detecting its two dimensions and then calculated by the following formula: V (tumour volume: mm^ 3^) = 0.5 × [W (width: mm)]^ 2^ × L (long diameter: mm). Four weeks later, the mice were sacrificed, and the xenograft tumour tissue was weighed. These tumour tissues were then fixed for further histological analysis. The immunohistochemistry procedure was performed as previously reported, and the percentages of stained area were calculated using ImageJ software 29. All programmes were authorized by the Institutional Animal Care and Use Committee of Xi'an Jiaotong University.

### Statistical analysis

To avoid systemic errors, each experiment was repeated more than three times. The results are displayed as the mean ± standard deviation. Student's t-test or one-way ANOVA (one-way analysis of variance) followed by the LSD post hoc test was conducted to compare the differences between two groups or more than two groups, respectively, with SPSS (SPSS 18.0; SPSS Inc., Chicago, IL, USA). A *P* value<0.05 was considered to be statistically significant.

## Results

### The level of miR-4319 expression was depressed in HCC compared with that in noncancerous tissues and correlated with adverse prognostic features

**Figure 1:**
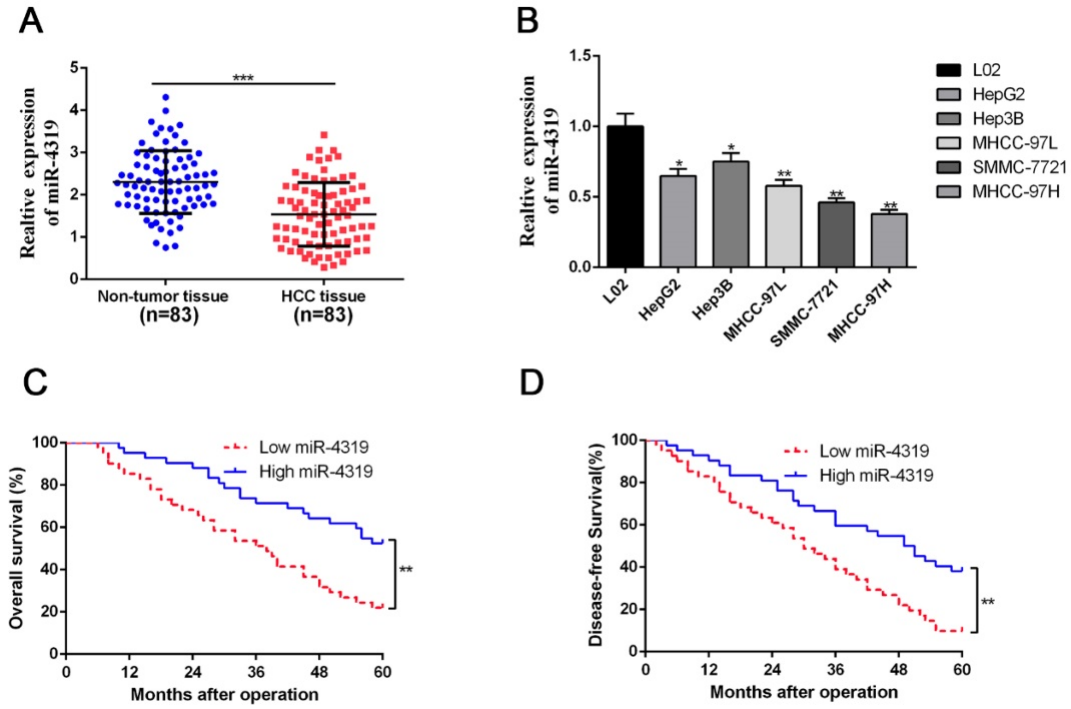
** MiR-4319 is reduced in HCC and predicts poor prognosis. (A)** The expression of miR-4319 was reduced in 83 HCC tissues compared to adjacent noncancerous tissues as determined by qRT-PCR. P < 0.0001 by t-test. **(B)** The differences in miR-4319 expression among HCC cell lines (HepG2, Hep3B, MHCC97L, SMMC-7721 and MHCC97H) and the human hepatocyte cell line (LO2). n=3 independent experiments, *P < 0.05 by ANOVA. **(C-D)** The HCC patients were divided into miR-4319 low-expression (n=41) and miR-4319 high-expression groups (n=42), with the median value of miR-4319 expression as a cut-off value. The overall survival and disease-free survival of HCC patients in the miR-4319 low-expression group were poorer than those in the high-expression group. P < 0.01 by log-rank test.

Due to the unclear biological role of miR-4319 in HCC, we first performed qRT-PCR analysis to examine its expression level in 83 pairs of HCC samples and corresponding pericarcinomatous tissues. The expression level of miR-4319 was markedly reduced in HCC samples in comparison to that in the corresponding adjacent nontumour tissues (P <0.01, Figure 1A). As shown in Table 1, the depression of miR-4319 was related to large tumour size (≥ 5 cm; P=0.017), high histological grade (Edmondson-Steiner grade III + IV; P=0.031) and venous invasion (P=0.001). Likewise, the expression of miR-4319 was obviously lower in the group of HCC cell lines compared to in the physiological liver cell line LO2 (P < 0.05, Figure 1 B). We selected MHCC-97H (relatively low expression of miR-4319) and Hep3B (relatively high expression of miR-4319) for further experiments. Furthermore, the overall survival and disease-free survival of HCC patients in the miR-4319 low-expression group was poorer than that of patients in the high-expression group (Figure 1C-D).

### MiR-4319 inhibits cell proliferation and accelerates apoptosis in HCC cells

**Figure 2:**
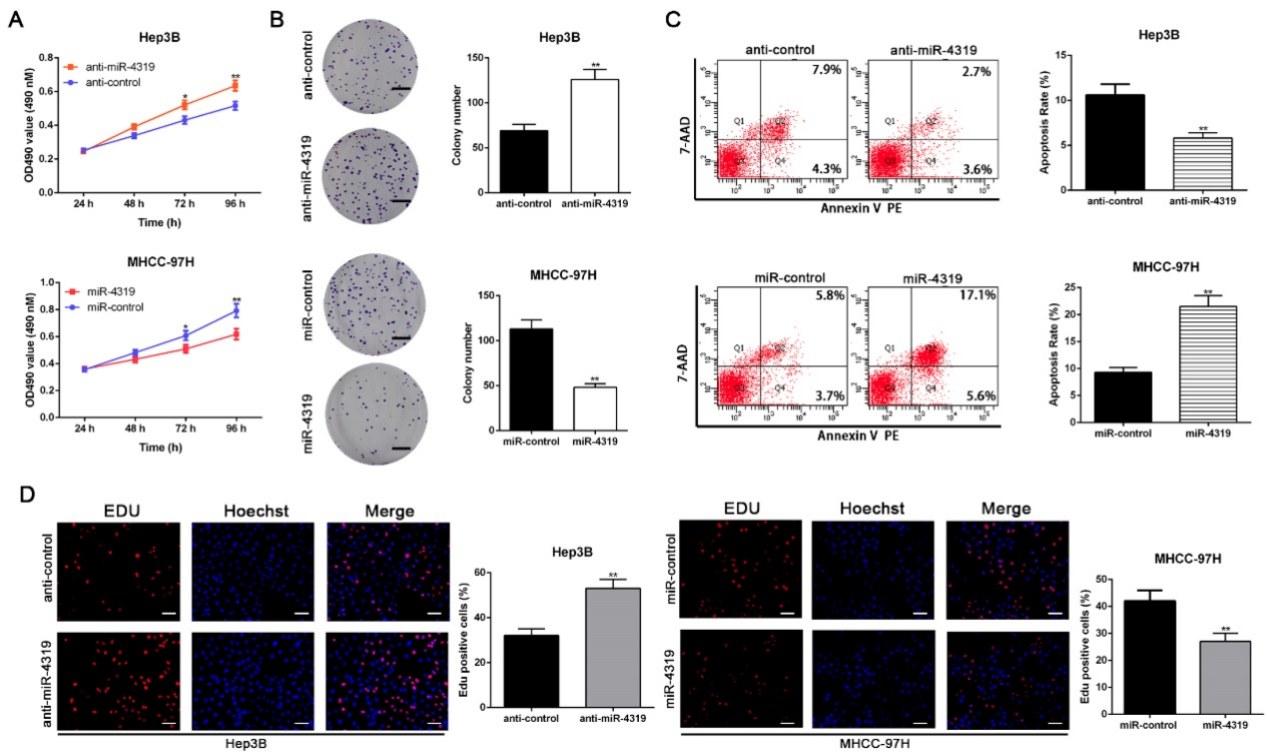
** MiR-4319 modulates HCC cell proliferation and apoptosis.** Hep3B cells transfected with miR-4319 inhibitor and MHCC-97H cells transfected with miR-4319 mimics were subjected to further experiments. Downregulation of miR-4319 promoted cell viability **(A)**, colony formation **(B)** and proliferation **(D)** and impeded apoptosis **(C)** in Hep3B cells, while overexpression of miR-4319 repressed cell viability **(A)**, colony formation (B) and proliferation **(D)** and accelerated apoptosis **(C)** in MHCC-97H cells. n=3 independent experiments, **P < 0.01 by t-test. Magnification of EdU is ×200, and scale bars = 50 μm. The scale bars of colony formation =1 cm.

To disclose whether miR-4319 plays a specific role in HCC progression, a series of functional experiments were conducted using a lentivirus system to stably overexpress MHCC-97H cells and knockdown Hep3B cells (Figure S1A-B, P<0.05). As evident from the colony formation assay, MTT assay and EdU incorporation assay, the over-expression of miR-4319 remarkably suppressed the proliferation of MHCC-97H cells in comparison to that of control cells (P<0.01, Figure 2A, 2B and 2D). Conversely, miR-4319 depletion in Hep3B cells enhanced proliferation with elevated cell colonies, increased the O.D value of MTT assay and upregulated the proportion of Edu-positive cells (P<0.01, Figure 2A, 2B and 2D). Additionally, miR-4319 overexpression prominently induced apoptosis of MHCC-97H cells, whereas Hep3B cells with miR-4319 depletion had a lower percentage of apoptotic cells than did the control cells (P < 0.01, Figure 2C). Collectively, these data illustrated that miR-4319 inhibits cell proliferation and accelerates apoptosis in HCC cells.

### Downregulation of miR-4319 induces EMT and confers cancer stem properties in HCC

**Figure 3:**
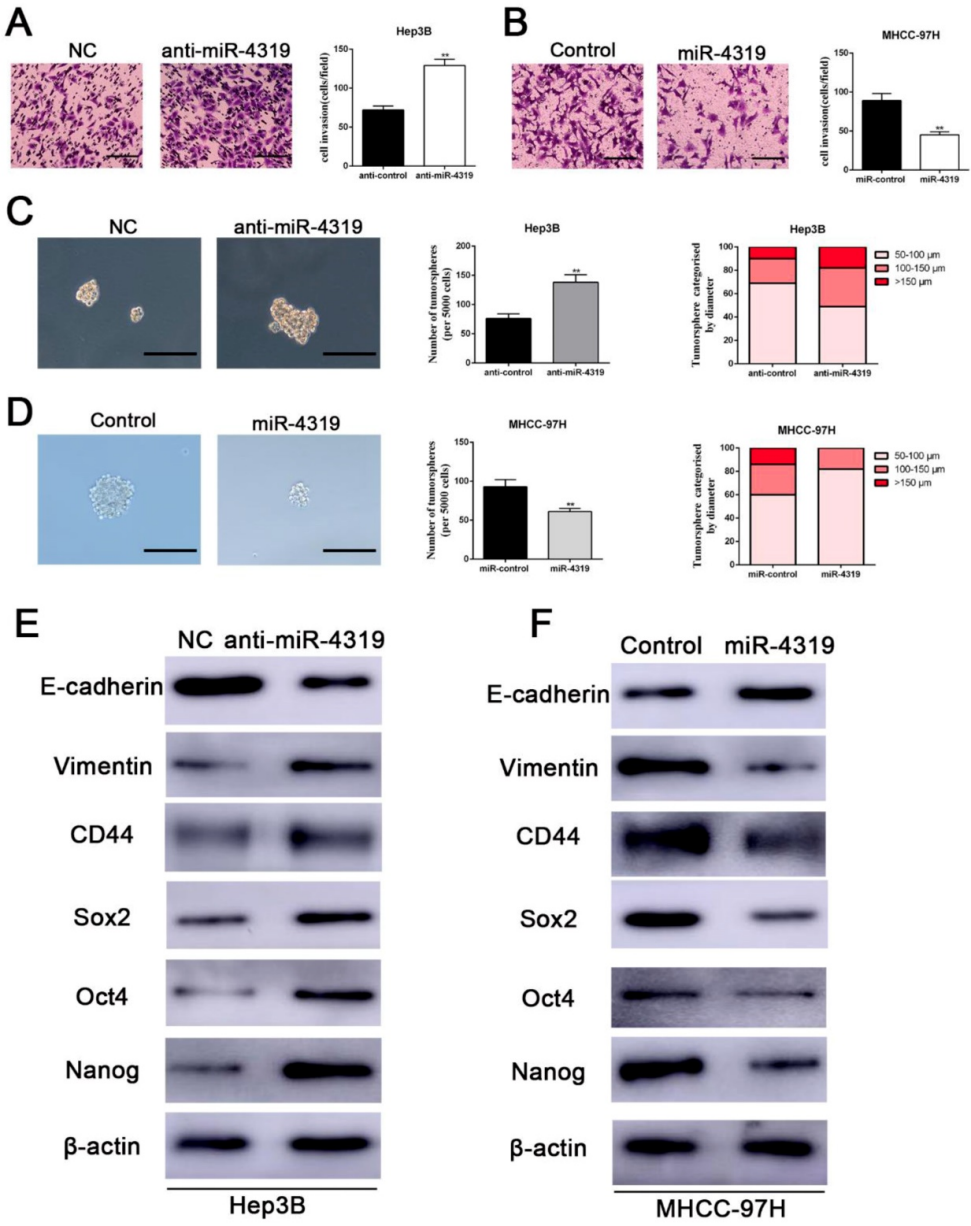
** Downregulation of miR-4319 induces EMT and confers cancer stem properties in HCC. (A)** Transwell-invasion and tumoursphere formation assays revealed that downregulation of miR-4319 enhanced invasive ability, increased the number of tumourspheres and elevated the percentage of tumourspheres with larger diameters **(C)** in Hep3B cells, while overexpression of miR-4319 inhibited cell invasion **(B)**, decreased the number of tumourspheres and elevated the percentage of tumourspheres with smaller diameters **(D)** in MHCC-97H cells. The results of western blot analysis **(E-F)** also showed that downregulation of miR-4319 induced EMT and facilitated the expression of cancer stemness markers (CD44, Sox2, Oct4, Nanog) in Hep3B cells, while overexpression of miR-4319 in MHCC-97H cells restrained EMT and cancer stemness. n=3 independent experiments, **P < 0.01 by t-test. The scale bars of transwell assay=50 μm. Magnification of tumoursphere is ×200, and scale bars = 50 μm.

We next examined whether the downregulation of miR-4319 in HCC also impacts EMT and tumour mammosphere formation. Silencing miR-4319 in Hep3B cells increased the invasive capacities of Hep3B cells, as confirmed by the Matrigel invasion assay (P < 0.01, Figure 3A). Moreover, tumoursphere formation, which is recognized as a representative trait of CSCs, was assayed to assess the sphere-forming capacity of HCC cells. The results displayed that both the number and the diameter of tumourspheres in Hep3B cells were greatly enhanced after knockdown of miR-4319 in Hep3B cells (Figure 3C). However, the Matrigel invasion assay and the tumoursphere formation assay indicated that the invasive capacities and the tumoursphere formation abilities of miR-4319-overexpressing MHCC-97H cells were repressed compared with those of control cells (Figure 3B, 3D). Moreover, the results of western blotting showed that the downregulation of miR-4319 in Hep3B cells induced the expression of EMT and CSC markers, as revealed by decreased E-cadherin expression and increased vimentin, CD44, CD133, CD90, EPCAM, Sox2, Oct4, and Nanog expression (Figure 3E and Figure S2). Analogously, overexpression of miR-4319 in MHCC-97H cells restrained the expression of EMT and CSC markers (Figure 3F and Figure S2). Thus, these results suggest that the downregulation of miR-4319 induces EMT and confers cancer stem properties in HCC.

### MiR-4319 suppresses tumourigenesis *in vivo*

**Figure 4:**
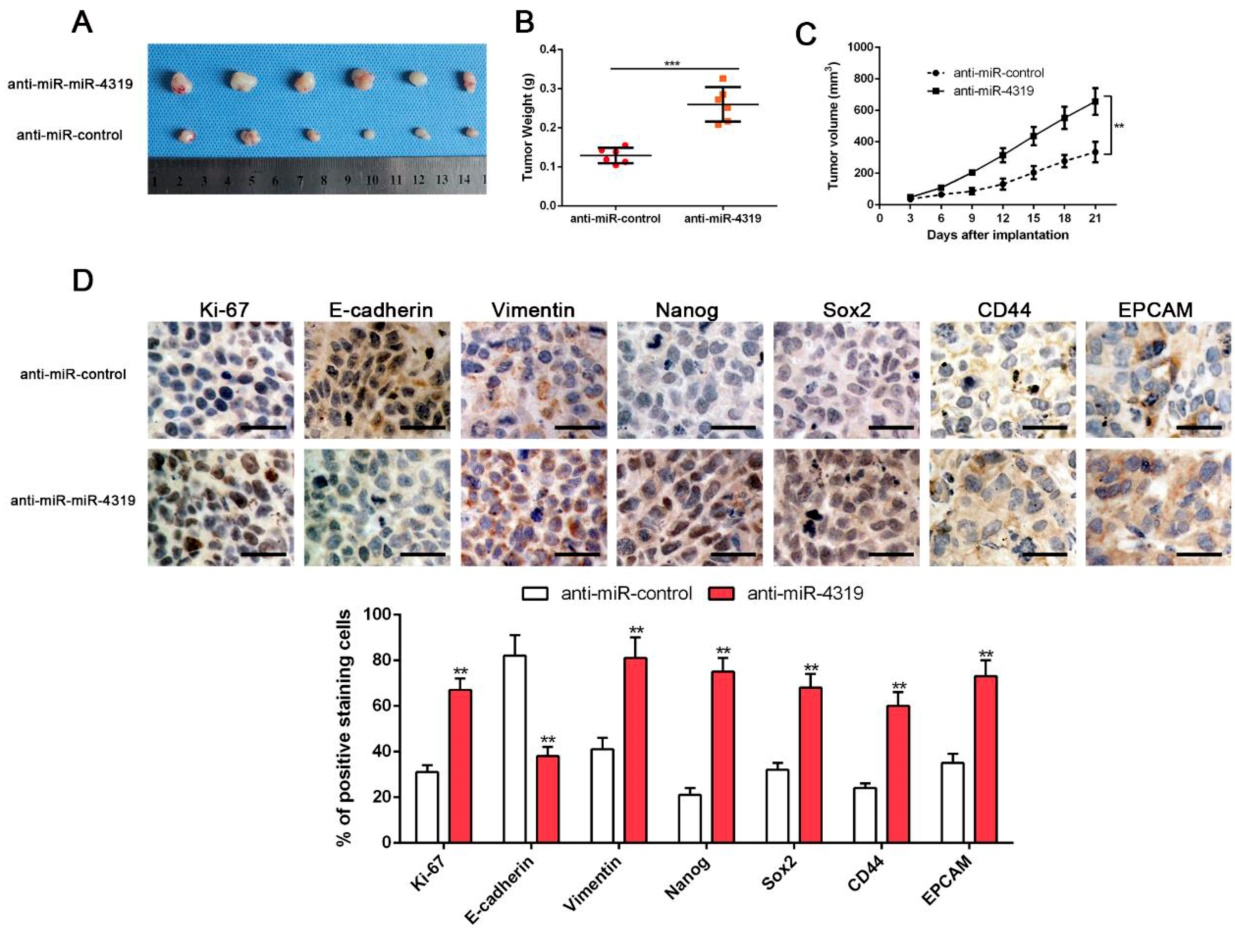
** MiR-4319 suppresses tumourigenesis *in vivo*. Stable miR-4319-depleting Hep3B cells were generated by infection with a lentiviral vector, and miR-4319-depleting Hep3B cells and control vector cells were implanted into nude mice via subcutaneous injection. (A)** Representative images of HCC xenografts from depleting Hep3B cells and control vector cells. **(B)** The tumour weight in the miR-4319-depleting Hep3B group was significantly heavier than that in the control group. n=6, ***P < 0.001 by t-test. **(C)** The tumour volume in the miR-4319-depleting Hep3B group (n=6) was obviously larger than that in the control group (n=6). **P < 0.01 by ANOVA. **(D)** The percentage of Ki-67, vimentin, Nanog Sox2, CD44 and EPCAM staining in tumour cells in the miR-4319-depleted Hep3B group was prominently higher than that in the control group, while the percentage of E-cadherin-stained tumour cells in the miR-4319 knockdown group was prominently lower than that in the control group. n=3 fields of 6 tissue sections, **P < 0.01 by t-test. Scale bar: 50 μm.

CSCs display stem cell-like properties, including self-renewal, tumour initiation, proliferation, invasion and metastasis 10, 30. Therefore, we investigated whether miR-4319 could repress* in vivo* tumourigenesis by subcutaneously injecting Hep3B cells with or without depletion of miR-4319. Hep3B cells with anti-miR-4319 generated a higher tumour weight and volume in comparison to those of the control group (Figure 4A-C). Moreover, immune-histochemistry staining and semiquantification analysis of the IHC data showed that subcutaneous tumours arising from depletion of miR-4319 in Hep3B cells increased the expression of EMT (E-cadherin and vimentin) and CSC (Nanog, Sox2, CD44 and EPCAM) markers in comparison to that in the control group (Figure 4D). It is worth noting that miR-4319 suppresses tumourigenesis and cancer stemness* in vivo*.

### FOXQ1 is identified as a direct target of miR-4319

**Figure 5:**
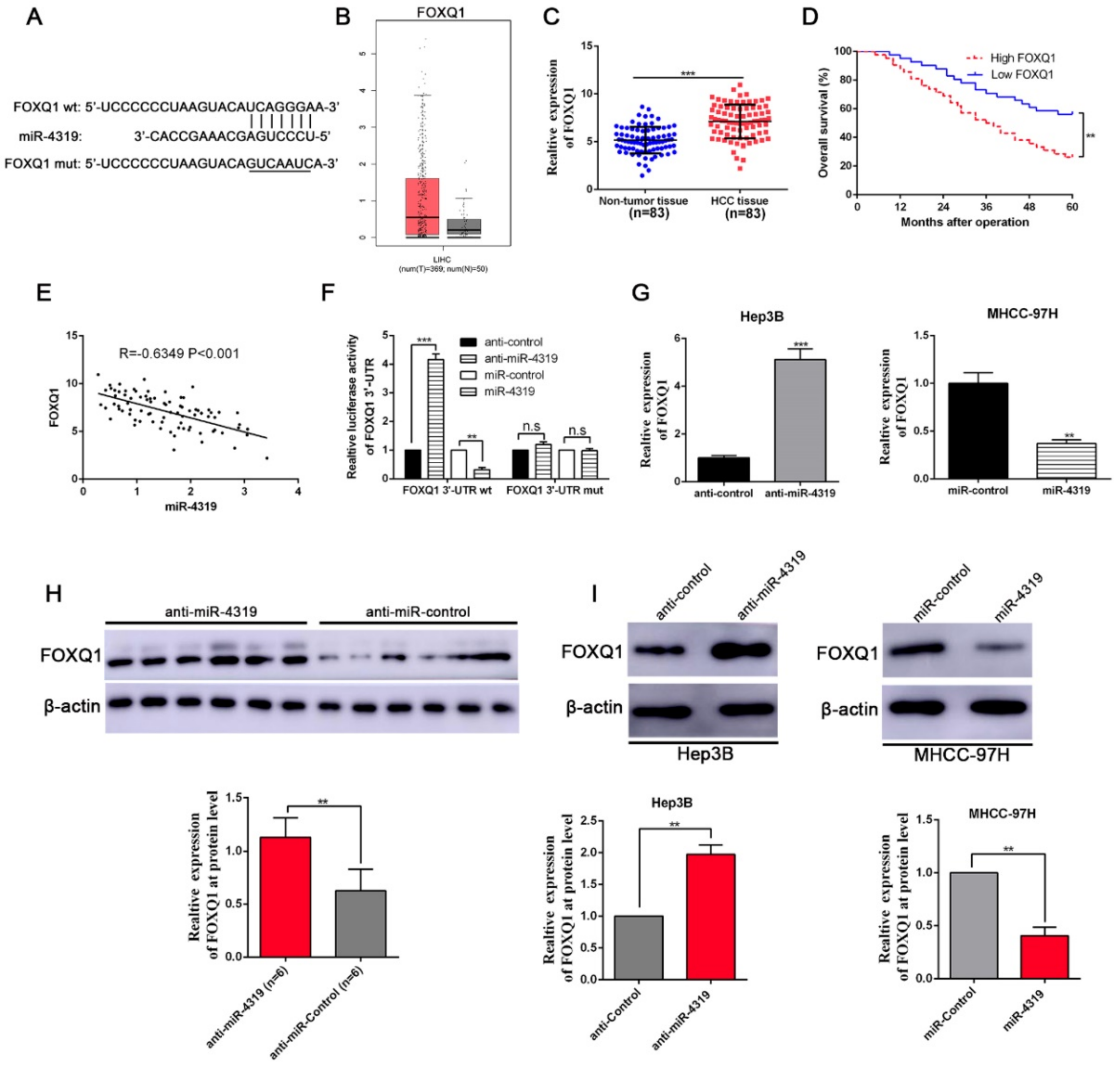
** FOXQ1 is a direct target of miR-4319. (A)** The putative binding sequences between miR-4319 and the 3'-UTR of FOXQ1 mRNA are shown. Mutated sequences in the 3'-UTR of FOXQ1 mRNA are underlined. **(B)** The expression pattern of FOXQ1 in HCC was derived from GEPIA. **(C)** The expression of FOXQ1 was increased in 83 HCC tissues compared to that in adjacent noncancerous tissues as determined by qRT-PCR. P < 0.0001 by t-test. **(D)** The HCC patients were divided into FOXQ1 low-expression (n=41) and miR-4319 high-expression (n=42) groups, with the median value of miR-4319 expression as a cut-off value. The overall survival of HCC patients in the FOXQ1 high-expression group was poorer than that in the low-expression group. P < 0.01 by log-rank test. **(E)** An inverse correlation between miR-4319 and FOXQ1 mRNA expression was observed in 83 HCC samples. P < 0.001 by Spearman's correlation test. **(F)** Overexpression of miR-4319 decreased while downregulation of miR-4319 increased the luciferase activity of vectors containing wt 3'-UTR of FOXQ1 rather than mut 3'-UTR of FOXQ1 in HEK-293T cells. n=3 independent experiments, **P < 0.01 by t-test. (G and I) miR-4319 overexpression reduced the levels of FOXQ1 mRNA and protein in MHCC-97H cells, while miR-4319 knockdown increased FOXQ1 abundance in Hep3B cells. n=3 independent experiments, **P < 0.01, *** P < 0.001 by t-test. (H) The expression of FOXQ1 in xenograft tissues. Xenograft tissues arising from the miR-4319 knockdown group (n = 6) and the control group (n = 6) were subjected to immunoblotting analysis to detect FOXQ1 expression at the protein level.

To uncover the underlying mechanisms by which miR-4319 impeded cell proliferation and repressed EMT and cancer stem traits in HCC, we performed bioinformatics analysis to predict the potential targets of miR-4319. Based on starBase V3.0 and TargetScan algorithms, FOXQ1, frequently reported as an oncogene to participate in the malignant progression of a variety of cancers^31^, was selected as a candidate interacting target of miR-4319 (Figure 5A). Previous studies have identified that FOXQ1 promotes proliferation, EMT, and cancer stemness^32^; thus, we first examined the expression of FOXQ1 in HCC. According to the data derived from GEPIA 33 and 83 paired samples of HCC and corresponding pericarcinomatous tissues, the expression level of FOXQ1 was elevated in HCC (Figure 5B-C). The OS and DFS of HCC patients in the FOXQ1 high-expression group were poorer than those in the low-expression group (displayed in Figure 5D and Figure S1C). As shown in Table 1, FOXQ1 overexpression was correlated with high histological grade (Edmondson-Steiner grade III + IV; P=0.029), late tumour stage (TNM stage III + IV; P=0.027) and venous invasion (P=0.032). Furthermore, we determined that there was a negative association between miR-4319 and FOXQ1 mRNA by Spearman's analysis in HCC samples (P<0.05, Figure 5E). Notably, the dual-luciferase reporter assay illuminated that enhanced expression of miR-4319 effectively repressed the relative luciferase activity of wild-type (wt) FOXQ1 instead of the mutant (mt) FOXQ1 in HEK-293T cells (P<0.05, Figure 5F). The expression of FOXQ1 and the potential downstream effectors of FOXQ1, Sox12 34 (sex determining region Y-box 12) and NDRG1 31 (N-myc downstream-regulated gene 1), were remarkably reduced at the protein levels in MHCC-97H cells with miR-4319 overexpression but enhanced in Hep3B cells with miR-4319 depletion (Figure 5G and 5I, Figure S3). Interestingly, we found that depletion of FOXQ1 in MHCC-97H cells reduced the expression of Sox12 and NDRG1, whereas overexpression of FOXQ1 in Hep3B cells enhanced the expression level of Sox12 and NDRG1 (Fig S3). Additionally, the expression of FOXQ1 protein in tumour tissues from miR-4319 knockdown-injected mice was significantly higher than that in control mice (Figure 5H). Altogether, FOXQ1 was a direct target of miR-4319.

### Restoration of FOXQ1 expression partially reversed the miR-4319-induced biological effects on HCC cells

**Figure 6:**
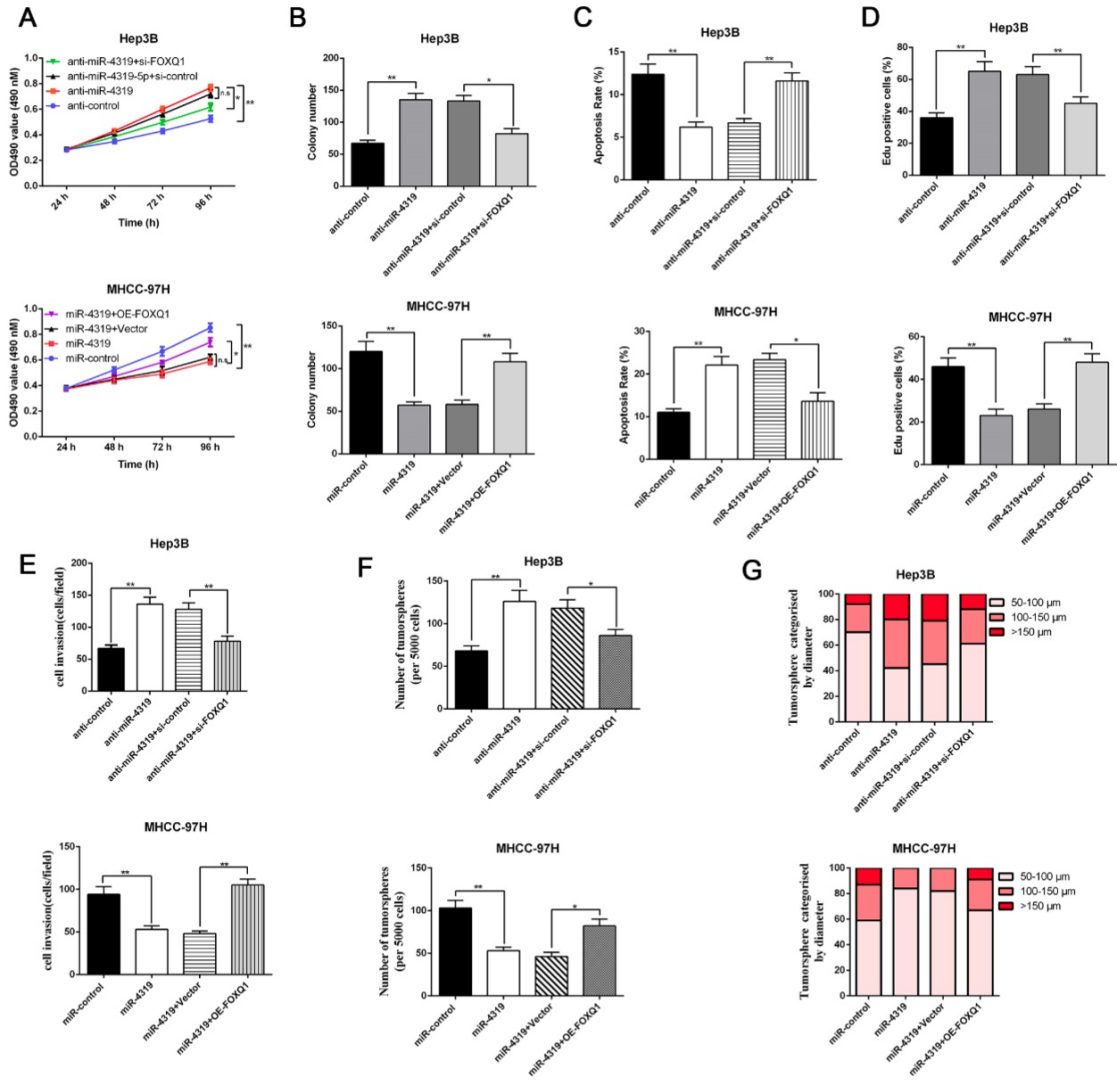
** Restoration of FOXQ1 attenuates the tumour suppressive effects of miR-4319.** MTT assay **(A)**, colony formation assay **(B)** and EdU assay **(D)** revealed that the proliferation of Hep3B cells was enhanced by miR-4319 depletion and subsequently rescued by FOXQ1 knockdown, while overexpression of FOXQ1 abolished the inhibition of proliferation induced by miR-4319 overexpression in MHCC-97H cells. (C) The apoptosis assay uncovered that the apoptosis of Hep3B cells was reduced by miR-4319 depletion and subsequently rescued by FOXQ1 knockdown, while overexpression of FOXQ1 abolished the promotion of apoptosis induced by miR-4319 overexpression in MHCC-97H cells. **(E-G)** Invasion and tumoursphere formation were increased by miR-4319 depletion and subsequently rescued by FOXQ1 restoration, while invasion, and oncosphere formation ability were dramatically impaired by overexpression of miR-4319. n=3 independent experiments, **P < 0.01 by t-test.

**Figure 7:**
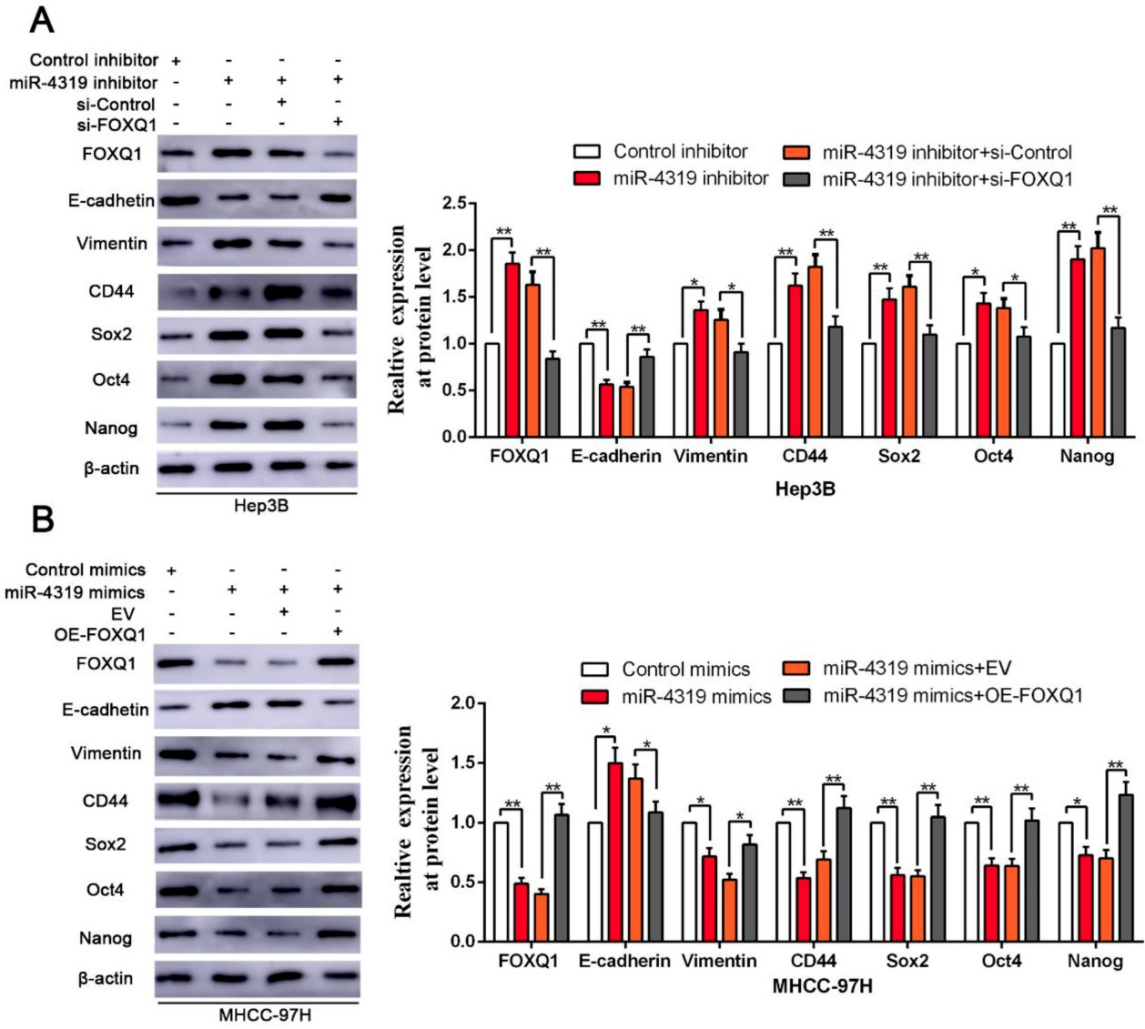
** Reintroduction of FOXQ1 expression partially reversed the miR-4319-induced inhibition on EMT and cancer stemness of HCC cells. (A-B)** FOXQ1 knockdown in miR-4319-suppressive Hep3B cells partly ceased the promotion of anti-miR-4319 on EMT and cancer stemness, whereas reintroduction of FOXQ1 rescued the miR-4319-induced inhibition on EMT (E-cadherin and Vimentin) and the CSC markers (CD44, Sox2, Oct4 and Nanog) of MHCC-97H cells. n=three independent experiments, **P < 0.01 by t-test.

Since FOXQ1 contributes to cancer stem traits and tumourigenesis in HCC, we aimed to investigate whether FOXQ1 mediated the biological effects of miR-4319 in HCC. FOXQ1 was overexpressed in miR-4319-overexpressing MHCC-97H cells (P<0.05, Figurre 6A), revealing that FOXQ1 restoration abolished the potency of miR-4319, leading to a remarkable increase of cell viability, proliferation and colony formation and a decrease in apoptosis (P<0.05, Figure 6A-D). The Matrigel invasion assay and the tumoursphere formation assay verified that FOXQ1 restoration attenuated the suppressive role of miR-4319 in the invasive capacities and tumoursphere formation abilities of MHCC-97H cells (P < 0.01, Figure 6E-G). Western blot analysis also indicated that reintroduction of FOXQ1 rescued the miR-4319-induced inhibition of the expression of EMT and CSC markers in MHCC-97H cells (P < 0.05, Figure 7A-7B and Figure S4A-S4B). In contrast, FOXQ1 knockdown in miR-4319-suppressive Hep3B cells partly ceased the potency of anti-miR-4319 on tumour cell proliferation, colony formation, apoptosis, invasion and cancer stem traits (P<0.05, Figure 6A-G, Figure 7A-7B and Figure S4A-S4B). Thus, the data indicated that FOXQ1 was a functional target of miR-4319 in HCC.

## Discussion

Hepatocellular carcinoma (HCC) commonly displays dismal outcomes because of its high possibility of metastatic relapse and chemotherapy resistance. The heterogeneity existing in tumours is responsible for the poor response to treatment 35, 36. Therefore, elucidating the molecular mechanisms of intratumoural heterogeneity in HCC is vital for the discovery of new therapeutic methods for improving the prognosis of patients. Of note, cancer stem cells (CSCs) existing in HCC may well explain the pathological properties of heterogeneity and recurrence 37. Herein, we demonstrated that the level of miR-4319 was remarkably decreased in HCC specimens and cells and that miR-4136 induced inhibition of the epithelial-mesenchymal transition and prevented cancer stemness of HCC through targeting FOXQ1. Thus, miR-4136, as a post transcriptional regulator, plays a profound role in suppressing the cancer stemness of HCC, which is in accord with findings that miR-4319 exerted an essential role as a tumour suppressor in the repression of cancer stemness in triple-negative breast cancer via downregulation of E2F2^22^.

It is well known that FOX family members play a pivotal role in cancer initiation and progression by modulating the cell cycle, DNA damage repair and cancer stem properties 38. Among FOX family members, FOXQ1 has also been reported to be upregulated and facilitate proliferation, EMT, and distant dissemination in a variety of human cancers, including HCC 34, 39, 40. Some studies have also unveiled an emerging role of FOXQ1 in modulating cancer stemness. For example, FOXQ1 maintains stemness and drives chemoresistance through its direct targets PDGFRα and β in breast cancer^32^; FOXQ1 also elevates tumour re-initiation in targeted organs and enhances the colonization of disseminated metastatic cells via LAMA4^41^. Moreover, FOXQ1 plays essential roles in mediating crosstalk between CAFs and HCC cells. CAFs induce expression of FOXQ1 in HCC cells, and N-myc downstream-regulated gene 1 (NDRG1) is subsequently trans-activated by FOXQ1 to enhance HCC initiation. Intriguingly, pSTAT6/C-C motif chemokine ligand 26 (CCL26) signalling is also modulated by the FOXQ1/NDRG1 axis, therefore recruiting CAFs to form a positive feedback loop in HCC 31.

During the past decade, miRNAs have emerged as major players in the complex network of gene regulation, and dysregulation of miRNA expression has been implicated in carcinogenesis 42. Previously published studies have highlighted that specific miRNAs contribute to HCC progression, and several of these can be used as biomarkers for diagnosis, prognosis, and metastasis predictions in HCC patients 40, 43. In fact, a few studies have shown that miRNAs may be able to regulate specific FOX genes in different cancers, including oesophageal cancer 44, hepatocellular carcinoma 45, and colorectal cancer 28; however, such an interaction between miRNAs and FOX proteins remains largely unclear and has not been interrogated in hepatocellular carcinoma. In this study, we corroborated through a series of gain- and loss-of-function assays that miR-4319 impeded cell proliferation, exacerbated cell apoptosis, dampened EMT and mitigated cancer stem traits. As a tumour suppressor, miR-4319 directly targets the 3-UTR of FOXQ1 and controls its expression in HCC. We also confirmed that FOXQ1 expression was enhanced in HCC tissues and negatively correlated with miR-4319 expression. Moreover, alteration of FOXQ1 levels could partially rescue the physiological roles of miR-4319 in HCC cells. Thus, our study highlights the miR-4319/FOXQ1 cascade as a potential therapeutic target for conquering HCC.

Collectively, this study shed light on the functional role and modulatory mechanism of miR-4319 in the malignant progression of HCC. We revealed that miR-4319 directly dampened the expression level of FOXQ1 to mitigate malignant progression of HCC through impeding cell proliferation, accelerating apoptosis, inhibiting epithelial-mesenchymal transition and preventing cancer stemness of HCC, which offered insight into the molecular mechanism underlying miR-4319 in HCC development. Moreover, the miR-4319/FOXQ1 cascade may be a novel target via dampening cancer stem traits to ameliorate heterogeneity and improve the treatment outcomes of HCC.

## Supplementary Material

Supplementary figures and tables.

## Figures and Tables

**Table 1:** Correlation between the clinicopathologic characteristics and miR-4319 and FOXQ1 expression in HCC (n = 83).

Clinical parameters	Cases	Expression level		*P* value	Expression level		*P* value
		MiR-4319^high^ (n=42)	MiR-4319^low^ (n=41)		FOXQ1^high^ (n=42)	FOXQ1^low^ (n=41)	
Age(years)							
<65 years	55	27	28	0.699	30	25	0.314
≥65 years	28	15	13		12	16	
Gender							
Male	71	37	34	0.503	36	35	0.964
Female	12	5	7		6	6	
Tumor size (cm)							
<5cm	38	26	12	0.017^*^	13	25	0.154
≥5cm	45	19	26		27	28	
Tumor number							
solitary	68	38	30	0.114	33	35	0.451
multiple	15	5	10		9	6	
Edmondson							
Ⅰ+Ⅱ	54	32	22	0.031^*^	20	34	0.029^*^
Ⅲ+Ⅳ	29	10	19		18	11	
TNM stage							
Ⅰ+Ⅱ	62	40	22	0.081	27	35	0.027^*^
Ⅲ+Ⅳ	21	9	12		15	6	
Capsular							
Present	58	30	28	0.756	31	27	
Absent	25	12	13		15	10	
Venous invasion							
Present	23	5	18	0.001^*^	16	7	0.032^*^
Absent	60	37	23		26	34	
AFP							
<400ng/ml	22	13	9	0.353	8	14	0.195
≥400ng/ml	61	29	32		32	29	
HBsAg							
positive	68	32	36	0.294	33	35	0.896
negative	15	6	9		7	8	

HCC, hepatocellular carcinoma; AFP, alpha-fetoprotein; TNM, tumor-node-metastasis. *Statistically significant.

## Abbreviations

HCC: hepatocellular carcinoma
CSCs: cancer stem cells
miRNAs: microRNAs
FOXQ1: Forkhead box Q1
EMT: epithelial-mesenchymal transition
NSCLC: non-small cell lung cancer
EdU: Ethynyl deoxyuridine
IHC: immunohistochemistry
MTT: 3-(4,5-Dimethylthiazol-2-yl)-2,5-diphenyltetrazolium bromide
ANOVA: one way analysis of variance
Sox12: sex determining region Y-box 12
NDRG1: N-myc downstream-regulated gene
