# Supplementary material for: MiR-4319 induced an inhibition of epithelial-mesenchymal transition and prevented cancer stemness of HCC through targeting FOXQ1
Source: Int J Biol Sci. 2019 Nov 15;15(13):2936–47. doi: 10.7150/ijbs.38000 (PMC6909970; doi:10.7150/ijbs.38000)
Supplement: Supplementary file 1 — Supplementary figures and tables. [file ijbsv15p2936s1.pdf]

Figure S1 The relative expression of miR-4319 and prognosis of FOXQ1in HCC. (A-B) Hep3B and MMCC-97H cells that were transfected with miR-4319 inhibitor or miR-4319 mimics, respectively, were detected by qRT-PCR for miR-4319 expression. n=3 independent experiments, **P < 0.01 by t-test. (C) The HCC patients were divided into FOXQ1 low-expression (n=41) and miR-4319 high-expression groups (n=42), with the median value of miR-4319 expression as a cut-off value. The disease-free survival of HCC patients in the FOXQ1 high-expression group was poorer than that of patients in the low-expression group. P < 0.01 by log-rank test.

Figure S2. Depletion of miR-4319 promotes the expression of cancer stemness markers in HCC. (A-B) Downregulaion of miR-4319 induced EMT and facilitated the relative expression of cancer stemness markers (CD44, Sox2, Oct4, Nanog) in Hep3B cells, while overexpression of miR-4319 in MHCC-97H cells restrained EMT and cancer stemness. n=three independent experiments, **P < 0.01 by t-test. (C-D) Depletion of miR-4319 in Hep3B cells also enhanced the expression of EPCAM, CD90 and CD133, whereas overexpression of miR-4319 in MHCC-97H cells repressed the expression of EPCAM, CD90 and CD133. n=three independent experiments, **P < 0.01 by t-test.

Figure S3. NDRG1 and Sox12, the potential downstream effectors of FOXQ1, was regulated by miR-4319. (A-B) anti-miR-4319 in Hep3B cells enhanced the expression level of Sox12 and NDRG1, while overexpression of miR-4319 in MHCC-97H cells reduced the expression of Sox12 and NDRG1. n=three independent experiments, **P < 0.01 by t-test.

Figure S4. Restoration of FOXQ1 reverses the inhibition of miR-4319 on cancer stemness in HCC. (A-B) EPCAM, CD90 and CD133 were also increased by miR-4319 depletion and subsequently rescued by FOXQ1 restoration in Hep3B cells; while overexpression of FOXQ1 rescued the miR-4319-induced inhibition on the expression of these CSC markers in MHCC-97H cells. n=three independent experiments, **P < 0.01 by t-test.

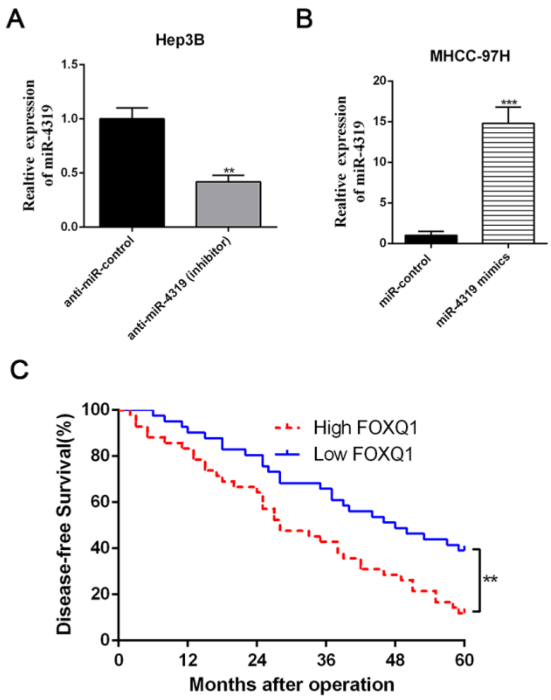

A
Hep3B
Realtive expression of miR-4319
1.5
1.0
0.5
0.0
**
anti-miR-control
anti-miR-4319 (inhibitor)
B
MHCC-97H
Realtive expression of miR-4319
20
15
10
5
0
***
miR-control
miR-4319 mimics
C
High FOXQ1
Low FOXQ1
Disease-free Survival(%)
100
80
60
40
20
0
**
0
12
24
36
48
60
Months after operation

Figure S1

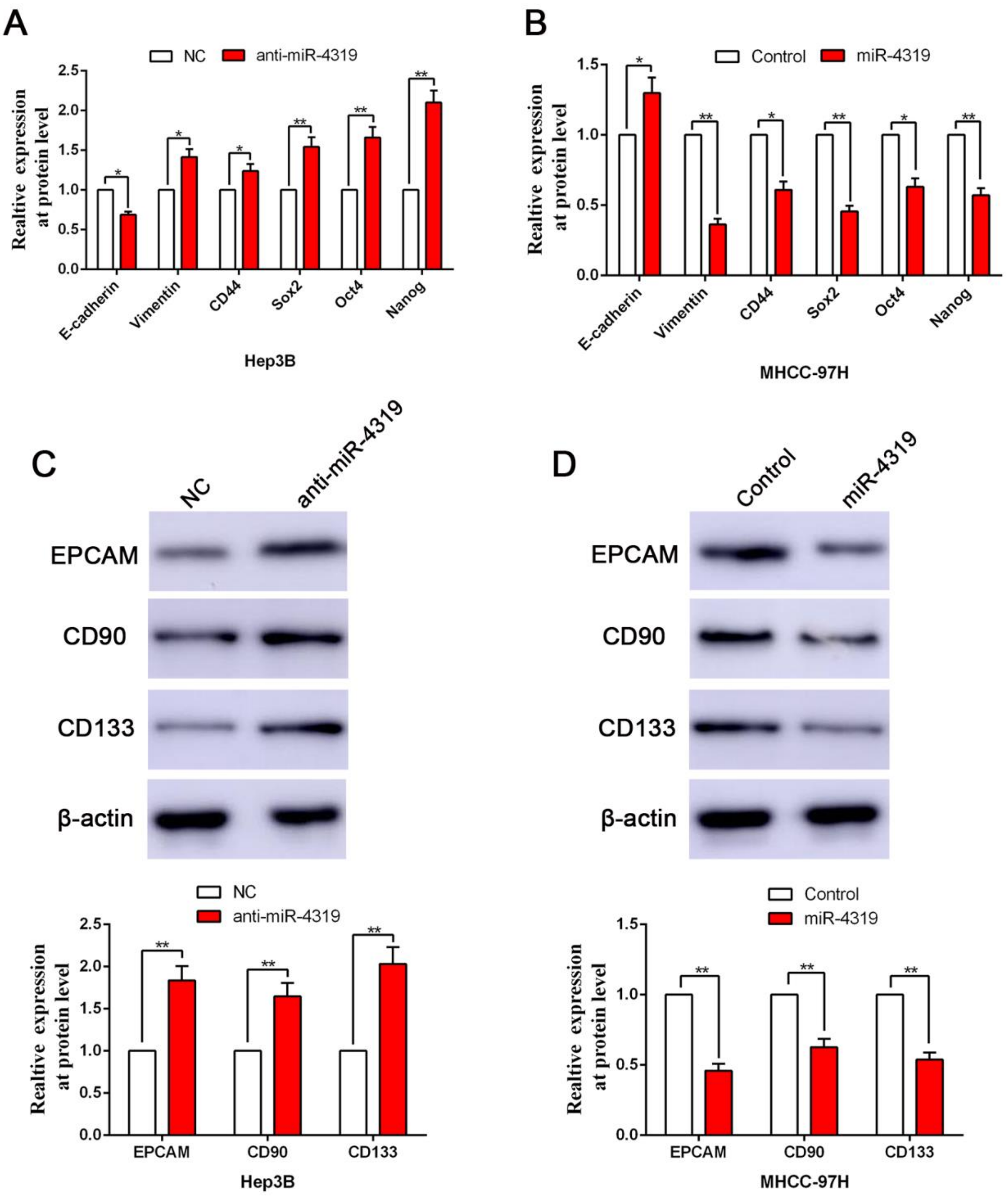
A
NC
anti-miR-4319
Realtive expression at protein level
E-cadherin
Vimentin
CD44
Sox2
Oct4
Nanog
Hep3B
B
Control
miR-4319
Realtive expression at protein level
E-cadherin
Vimentin
CD44
Sox2
Oct4
Nanog
MHCC-97H
C
NC
anti-miR-4319
EPCAM
CD90
CD133
β-actin
NC
anti-miR-4319
Realtive expression at protein level
EPCAM
CD90
CD133
Hep3B
D
Control
miR-4319
EPCAM
CD90
CD133
β-actin
Control
miR-4319
Realtive expression at protein level
EPCAM
CD90
CD133
MHCC-97H

Figure S2

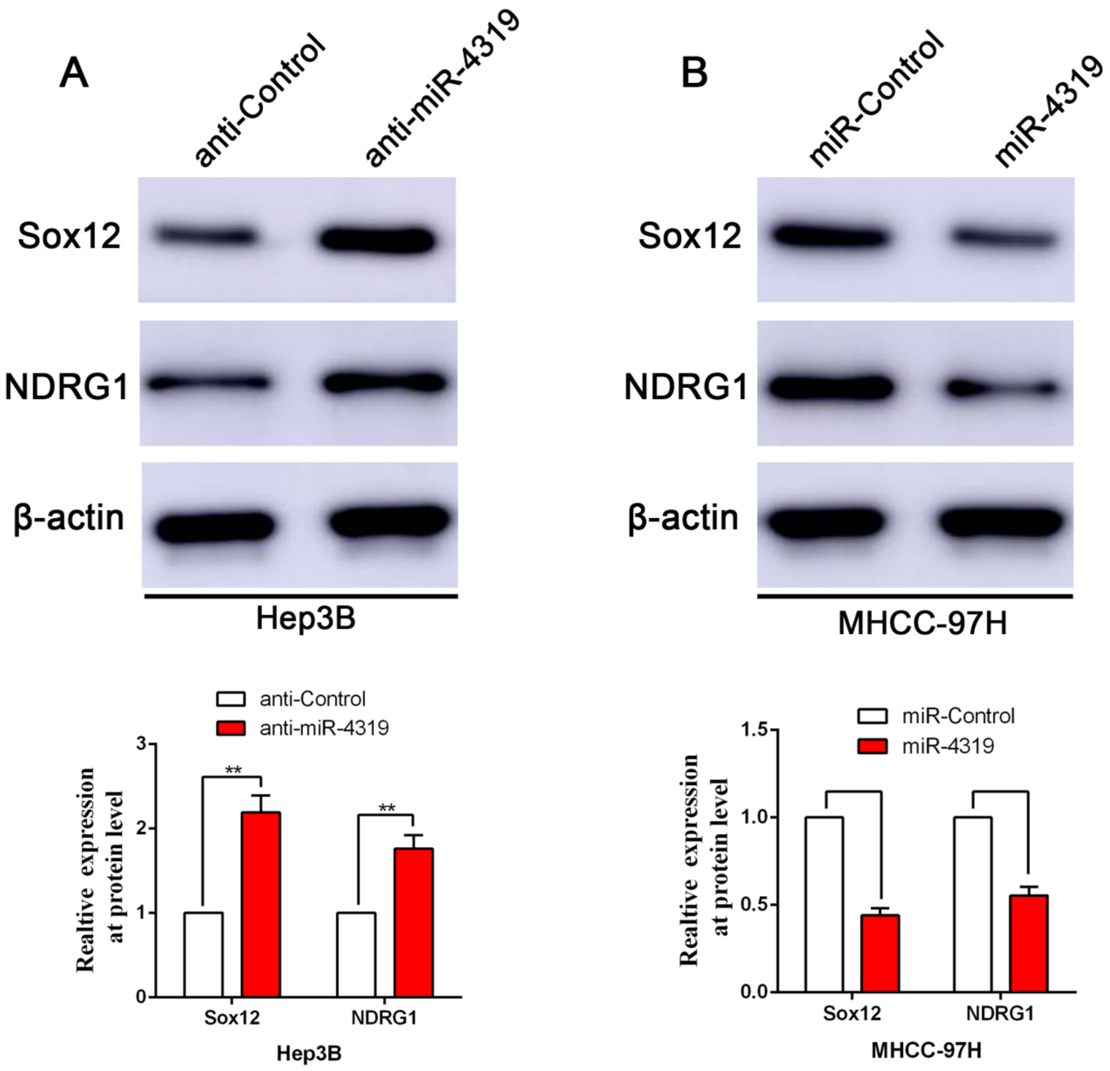
A
anti-Control
anti-miR-4319
Sox12
NDRG1
β-actin
Hep3B
B
miR-Control
miR-4319
Sox12
NDRG1
β-actin
MHCC-97H
anti-Control
anti-miR-4319
Realtive expression at protein level
3
2
1
0
**
**
Sox12
NDRG1
Hep3B
miR-Control
miR-4319
1.5
1.0
0.5
0.0
Realtive expression at protein level
Sox12
NDRG1
MHCC-97H

Figure S3

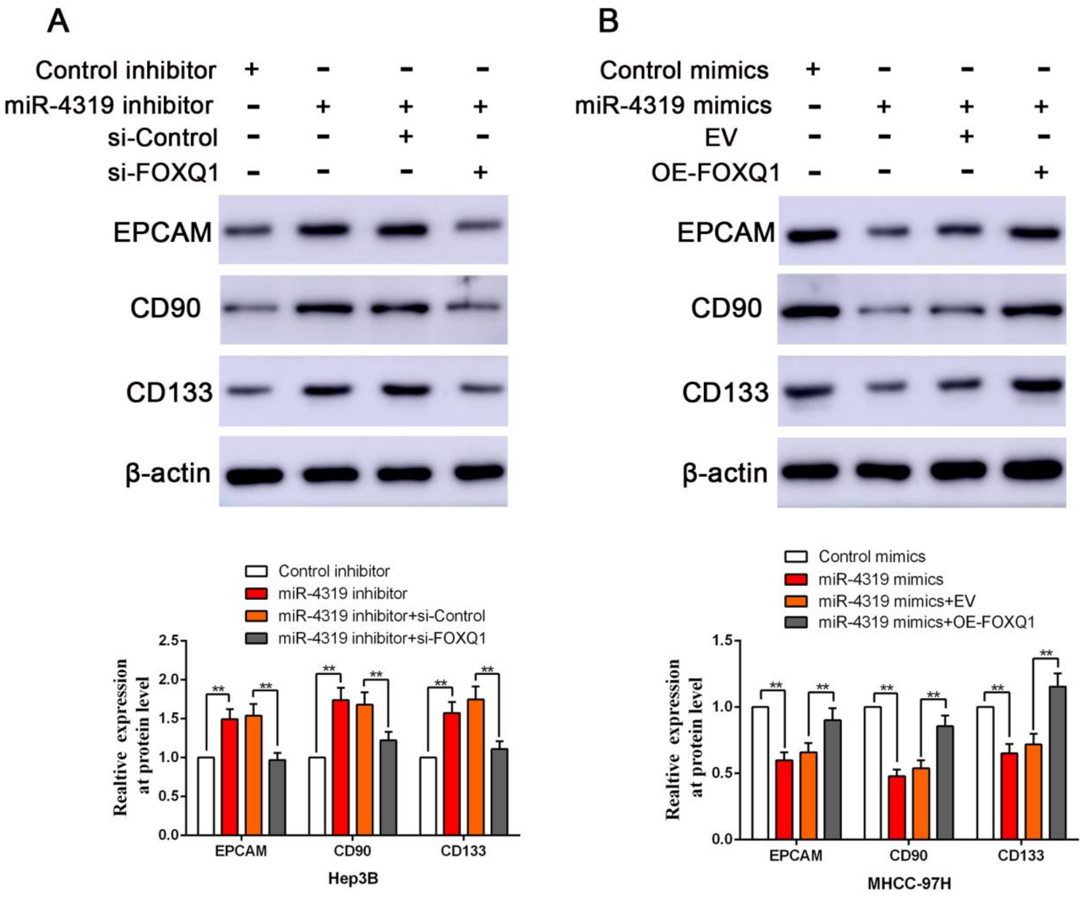

Figure S4

Supplementary Table 1. A list of the utilized primary antibodies

Antibody	Dilution & Use	Company
Rabbit anti-E-cadherin	1:1000 (WB)	Cell Signaling Technology
Rabbit anti-CD44	1:1000 (WB)	Abcam
Rabbit anti-vimentin	1:1000 (WB)	Cell Signaling Technology
Rabbit anti-FOXQ1	1:1000 (WB)	Abcam
Rabbit anti-Sox2	1:1000 (WB)	Abcam
Rabbit anti-OCT4	1:1000 (WB)	Abcam
Rabbit anti-Nanog	1:1000 (WB)	Abcam
Rabbit anti-ki-67	1:500 (IHC)	Abcam
Rabbit anti-E-cadherin	1:400 (IHC)	Cell Signaling Technology
Mouse anti-β-actin	1:10,000 (WB)	Sigma
Rabbit anti-vimentin	1:500 (IHC)	Cell Signaling Technology
Rabbit anti-EPCAM	1:1000 (WB)	Abcam

Rabbit anti-CD90	1:1000 (WB)	Abcam
Rabbit anti-CD133	1:1000 (WB)	Abcam
Rabbit anti-Sox12	1:500 (WB)	SAB (Signalway Antibody)
Rabbit anti-NDRG1	1:5000 (WB)	Abcam
Rabbit anti-Sox2	1:200 (IHC)	Abcam
Rabbit anti-OCT4	1:200 (IHC)	Abcam
